# Structural Determinants for Protein adsorption/non-adsorption to Silica Surface

**DOI:** 10.1371/journal.pone.0081346

**Published:** 2013-11-25

**Authors:** Christelle Mathé, Stéphanie Devineau, Jean-Christophe Aude, Gilles Lagniel, Stéphane Chédin, Véronique Legros, Marie-Hélène Mathon, Jean-Philippe Renault, Serge Pin, Yves Boulard, Jean Labarre

**Affiliations:** 1 Laboratoire de Radiolyse, SIS2M, IRAMIS and UMR3299 CEA-CNRS, Saclay, France; 2 Service de Biologie Intégrative et Génétique Moléculaire, iBiTec-S, FRE3377 CEA-CNRS-Université Paris-Sud, Saclay, France; 3 Laboratoire Analyse et Modélisation pour la Biologie et l'Environnement, UMR 8587 CNRS-Université Evry Val d'Essonne, Evry, France; 4 Laboratoire Léon Brillouin, IRAMIS and UMR12 CEA-CNRS, Saclay, France; 5 Laboratoire Structure et Dynamique par Résonance Magnétique, SIS2M, IRAMIS and UMR3299 CEA-CNRS, Saclay, France

## Abstract

The understanding of the mechanisms involved in the interaction of proteins with inorganic surfaces is of major interest in both fundamental research and applications such as nanotechnology. However, despite intense research, the mechanisms and the structural determinants of protein/surface interactions are still unclear. We developed a strategy consisting in identifying, in a mixture of hundreds of soluble proteins, those proteins that are adsorbed on the surface and those that are not. If the two protein subsets are large enough, their statistical comparative analysis must reveal the physicochemical determinants relevant for adsorption versus non-adsorption. This methodology was tested with silica nanoparticles. We found that the adsorbed proteins contain a higher number of charged amino acids, particularly arginine, which is consistent with involvement of this basic amino acid in electrostatic interactions with silica. The analysis also identified a marked bias toward low aromatic amino acid content (phenylalanine, tryptophan, tyrosine and histidine) in adsorbed proteins. Structural analyses and molecular dynamics simulations of proteins from the two groups indicate that non-adsorbed proteins have twice as many π-π interactions and higher structural rigidity. The data are consistent with the notion that adsorption is correlated with the flexibility of the protein and with its ability to spread on the surface. Our findings led us to propose a refined model of protein adsorption.

## Introduction

The adsorption of proteins on surfaces is a quasi-universal phenomenon of major physiological and toxicological significance. However, the mechanisms and structural determinants of protein/surface interactions are still unclear [1]. A crude description would assume that the main determinants of protein adsorption are electrostatic interactions on charged surfaces, and hydrophobic interactions on hydrophobic surfaces. This scheme is perfectly functional for chromatographic techniques [2], but fails to explain the “nonspecific adsorption” of proteins that occurs, for example, on biosensors, implants, etc [3,4]. The only way to prevent such adsorption is to expose the surface to a passivating protein like BSA, which will saturate all sites [5] or to an antifouling compound such as poly(ethylene glycol) [6]. The important question then is, rather than why a given protein is adsorbed, why should another protein not be adsorbed on a surface? In other words, is there a determinant of the relative sensitivity of proteins to nonspecific interactions? Obviously, the answer to this important question depends on the physical and chemical structure of the surfaces considered.

Owing to its omnipresence, silica (SiO_2_) is a reference material in the study of interactions of proteins with inorganic surfaces. The surface of crystalline silica is composed of silanol groups (Si-OH) and siloxane bridges (-Si-O-Si-). At pH higher than 3, the silanol groups tend to be deprotonated as Si-O^-^ leading to a negatively charged surface. Silica is used in a wide variety of applications. Nowadays, a growing utilization of silica is in the form of nanoparticles, mainly as an anti-agglomerant in drugs, creams, food, etc. Nanoparticles (NPs) are objects of nanometric size (< 100 nm). Thanks to their extremely low size, they have two major properties: (i) the ability to penetrate cells and reach toxicological targets or medical targets and (ii) a high surface/mass ratio that considerably amplifies the material’s surface available for interactions. These properties and the growing utilization of nanoparticules raise the question of their biological effects and health hazards. Upon contact with a biological fluid, e.g. when entering a cell or the blood stream, NPs are readily coated by proteins [7,8]. The nature of the proteins adsorbed on the NPs is a determining factor in the fate and biodistribution of the NPs in the organism (for review see [9]), a property that can be used in nanomedicine to design new carriers targeting specific organs or tissues [10,11]. In this context, a better understanding of adsorption mechanisms would be of great interest. Besides, some bio-surfaces, such as cell membranes or cell walls, are typically nanostructured [12,13], and probably present a higher degree of complexity [14] han standard NPs. Thus, studying protein-NPs interactions can be a relevant first approach to the more complex problem of understanding the interaction of proteins with extended nano-surfaces.

**Figure 1:**
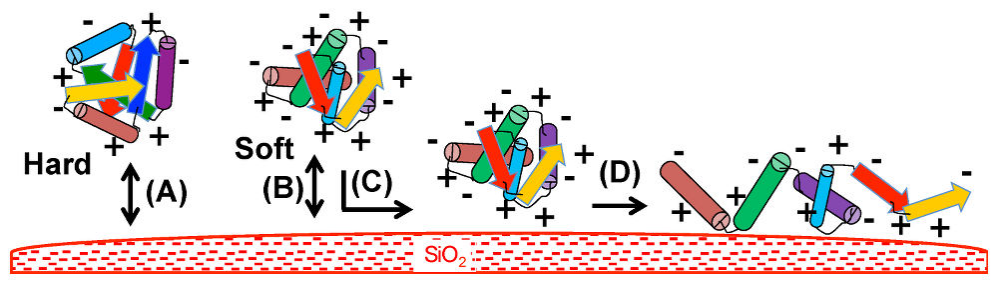
Scheme for the interaction of proteins with the silica surface. Interactions between the proteins and the surface are supposed to be initiated by electrostatic interactions between positively charged residues and the negative charges of the silica surface (A) and (B). This interaction remains transient and reversible in the case of “hard” proteins. In the case of “soft” proteins, the proteins can deform on the surface and establish other electrostatic contacts (C), which may lead to a spreading of the protein on the surface (D), a quasi-irreversible process.

The consensual view is that protein adsorption to silica (see schematic model in Figure 1) and similar metal oxides results from both (i) the electrostatic properties of the protein and (ii) its ability to induce structural deformation on the surface [15-18]. Electrostatics (ionic interactions and hydrogen bonds) is considered of major importance, at least for the first contacts (Figure 1A and 1B). In particular, basic amino acids (Arg and Lys, which are positively charged at neutral pH) are supposed to be essential to establish electrostatic interactions with the electronegative silica surface. This first step is considered reversible. The second step of the adsorption process is supposed to depend on the degree of “hardness-softness” of the proteins [1]. Rigid and tightly structured proteins (“hard” proteins) do not deform on the surface and are not prone to adsorption (Figure 1A). In contrast, proteins with weak internal cohesion (“soft” proteins) are more able to deform and structurally rearrange on the surface leading to increase the number of interactions and to a spreading of the protein on the surface (Figure 1C and 1D). During this conformational change, interactions may occur between the silica hydrophobic sites (e.g. siloxane bridges) and hydrophobic residues of the protein exposed to the surface. This second step is often considered irreversible. The adsorption process is mainly driven by an entropy gain arising from protein rearrangement and the release of water molecules in the bulk compartment. 

However, in spite of a wealth of studies on this question, the physicochemical parameters that could determine whether a protein is prone or resistant to adsorption have not been identified with precision. For example, it could be expected that a high content in positively charged amino acids or a low content of residues known to be important for protein “hardness” would favor adsorption. However, to our knowledge, no experimental approach has addressed the question of whether some specific amino acids promote or hinder the adsorption process. In addition, the protein adsorption studies have generally been performed on individual proteins, such as lysozyme and serum albumin [19,20]. Though this approach has greatly enhanced our understanding of the adsorption process for these model proteins, it does not allow easy comparisons or an overall vision of the phenomena involved.

To answer this fundamental question, we present in this work a proteomic strategy, which consists in identifying, in a large protein mixture, the proteins that bind to the surface and the proteins resistant to adsorption and in comparing the characteristics of the two protein subsets. This methodology has the advantage of throughput compared with most adsorption studies conducted on isolated, purified proteins. The strategy was tested with silica nanoparticles and our findings lead us to propose a refined model of protein adsorption.

## Results

### Preliminary characterizations

**Figure 2:**
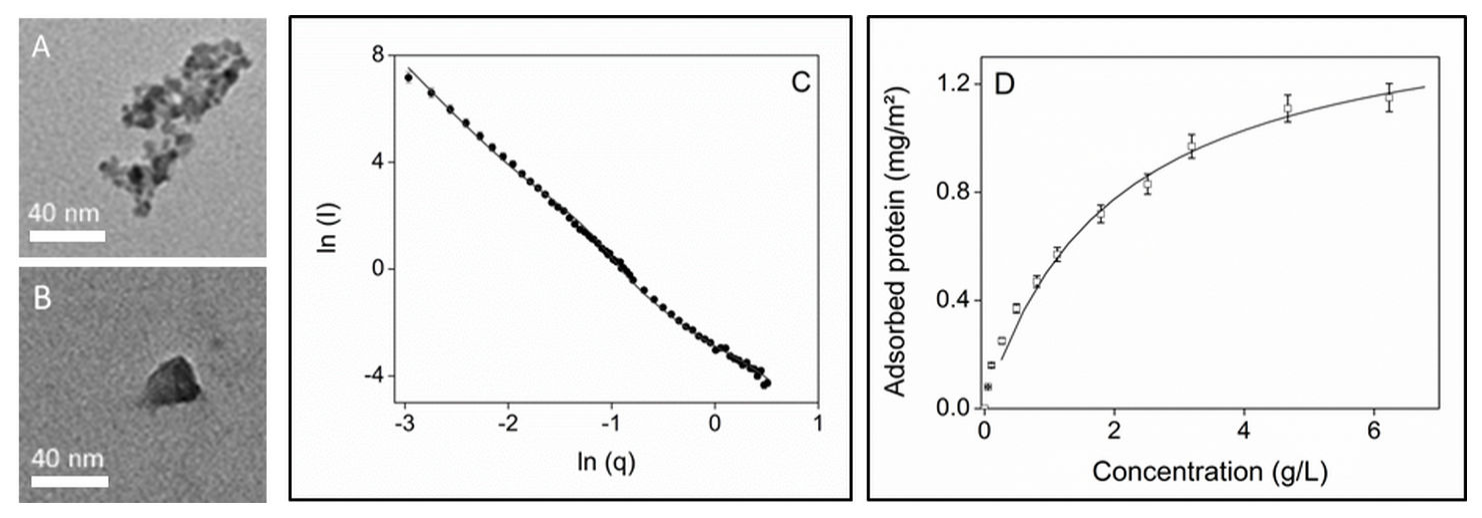
Characterization of silica nanoparticles used in the study. Transmission electron microscopy images of silica NPs: (A), small aggregated particles and (B), single large particle. (C) Small angle neutron scattering profiles of silica NPs; experimental data (■) and fitting by a particle size distribution (gray line). (D) adsorption isotherm of yeast proteins on silica NPs in PBS buffer; experimental data (□) and fitting by the Langmuir model (black line).

For this study, we used silica in the form of nanoparticles (NPs) as they provide a large specific surface for the subsequent analysis. The SiO_2_ NPs were suspended in a phosphate buffer containing 150 mM NaCl (PBS for phosphate buffered saline). NP size and shape were characterized by transmission electron microscopy (TEM) images (Figure 2A and 2B) and small-angle neutron scattering (SANS) experiments (Figure 2C). Both techniques show two distinct populations of particles, a major one composed of large spherical particles with a mean diameter of 26 ± 2 nm and a second one composed of smaller particles with a mean diameter of 5 ± 2 nm, possibly coming from the fragmentation of the larger ones. In aqueous solution, the NPs tend to form small aggregates of a mean size of 50 nm. NP surface area measured by gas adsorption was SBET = 168 m^2^/g. We determined a Zeta potential of -14.2 mV, which indicates a negatively charged surface at pH 7.4 in PBS buffer, consistent with the isoelectric point of pH 2 [21].

The yeast protein mixtures were prepared and labeled with sulfur 35 (see Materials and Methods section) and were first used to determine the overall adsorption isotherm at an NP concentration of 5 g/L and a protein concentration ranging from 0.25 g/L to 6.5 g/L (Figure 2D). The isotherm could be fitted by the Langmuir model [22]. The maximum quantity of proteins adsorbed on the surface was 1.58 mg/m^2^ under these conditions, which is similar to values obtained for the adsorption of pure proteins, such as serum albumin and lysozyme [19].

### Comparative two-dimensional electrophoresis

**Figure 3:**
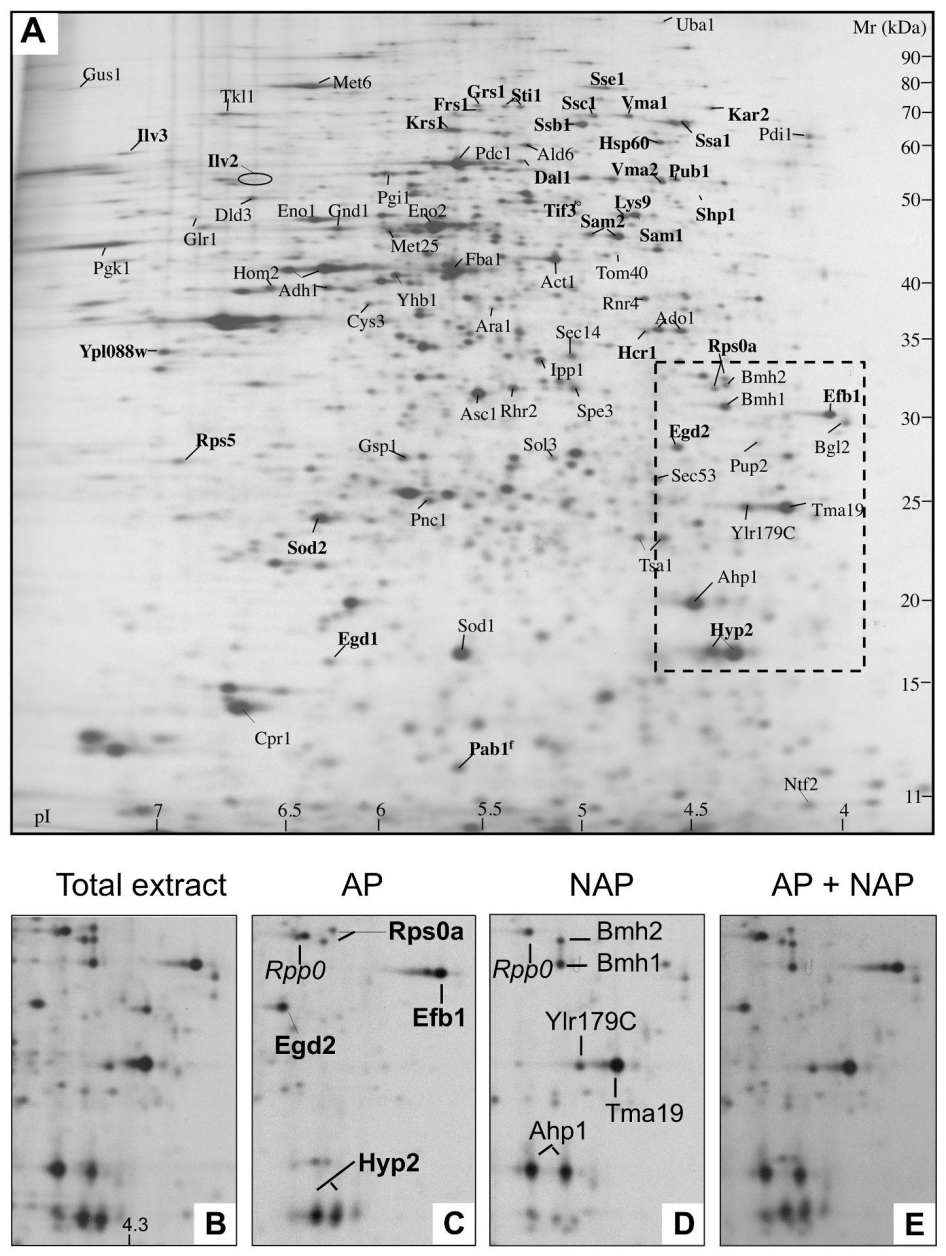
Autoradiograms of two-dimensional electrophoresis gels of ^35^S-labeled yeast proteins. (A) Reference map of total soluble yeast proteins; names of adsorbed and non-adsorbed proteins are indicated in bold and standard characters, respectively. The area of the gel focused in B, C, D and E (Mr 35-15 kDa, pI 4-4.6) is framed in the dotted line. (B) Total soluble proteins of the focused region. (C) Adsorbed proteins. (D) Non-adsorbed proteins. (E) Superposition of autoradiograms (C) and (D). Is also shown in italics a protein spot (Rpp0), which belongs to the intermediate group. Proteomic experiments were repeated 3 times with similar results.

For the next experiments designed to separate proteins that bind NPs from proteins resisting adsorption, we chose a concentration of the protein mixture of 1 g/L. At this concentration, 37% of the proteins were bound to the NPs and the rest (63%) remained in solution. Under these conditions, the surface covered by proteins is far from being saturated (0.55 mg/m^2^ of adsorbed proteins compared with a maximum capacity of 1.58 mg/m^2^), ensuring that unbound proteins are really resistant to adsorption. The ^35^S-labeled proteins bound to the NPs under these conditions were re-solubilized in the two-dimensional electrophoresis (2-DE) extraction buffer and run on 2-DE gels. In the same way, the labeled proteins remaining in the buffer (resistant to adsorption) were run on 2-DE gels (see Materials and Methods section). The autoradiographs of the gels revealed very different patterns of protein spots. This is illustrated in panels C (adsorbed proteins) and D (non-adsorbed proteins) of Figure 3, which show a selected part of the autoradiographs. A visual comparison of the 2 panels indicates a strong over-representation of some proteins (e.g. Efb1 and Egd2) in the adsorbed fraction and the presence of other proteins (e.g. Ahp1 and Tma19) exclusively in the non-adsorbed fraction. In this part of the gel, only one protein (Rpp0) belongs to both fractions, with equivalent amounts in each gel. It indicates a marked partition of the protein mixture into the adsorbed fraction and the non-adsorbed fraction. Figure 3 also shows the 2-DE gel of the total extract prior to adsorption (panel A and B) and the superimposition of autoradiographs of the two fractions (panel E) that reconstitute almost perfectly the 2-DE map of the total extract, indicating that very few proteins are lost in the experimental procedure (washing, desorption step, electrophoresis).

Based on this visual examination, we proposed to classify the proteins into 3 groups: the adsorbed proteins (APs), the non-adsorbed proteins (NAPs) and the proteins showing an intermediate behavior. To define these groups, we quantified the radioactivity present in each protein spot of each gel. After standardization of the data, the calculation of the ratio (AP/NAP) of ^35^S intensity for each protein allowed us to classify the proteins as APs, NAPs and intermediate proteins (see Materials and Methods section). These groups were composed respectively of 31, 46 and 24 proteins. The 31 APs and the 46 NAPs are listed in Table S1 with the ^35^S ratios and are indicated on the 2-DE maps of the total protein extract (Figure 3A).

### Statistical comparison of physicochemical parameters of APs versus NAPs

**Figure 4:**
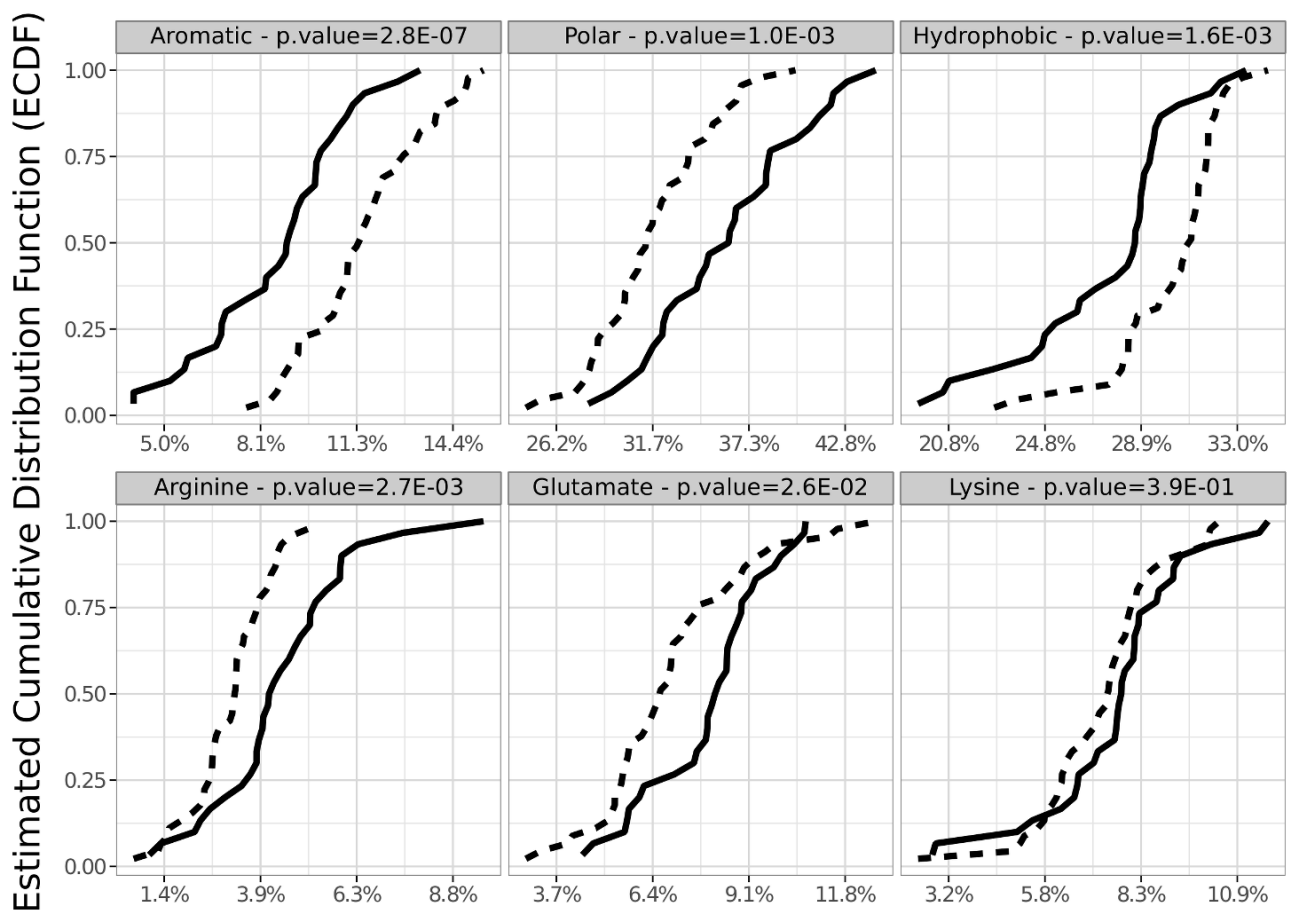
Cumulative distribution for selected parameters. The AP group is represented by a solid line and the NAP group by a dotted line. The parameters presented are aromatic amino acids (Phe + Tyr + Trp + His), polar amino acids, hydrophobic amino acids, Arginine, Glutamate and Lysine.

We undertook a statistical analysis based on amino acid sequences of the proteins of both subsets. We compared 46 features such as amino acid composition, hydrophobicity, polarity, size, isoelectric point, etc... that can be calculated based on amino acid sequences (listed in Table S2). It is clear that many of these parameters are partially linked, but the analysis was designed to identify the most significant ones, if any. For each feature we assessed the significance of the difference in between-group means using the Brunner-Munzel non-parametric test (see Materials and Methods section). Relevant features showing the greatest differences between the two groups are listed in Table 1, ordered by increasing p-values. For some selected parameters, the data are also shown as empirical cumulative distributions representative of the 2 sets of proteins (Figure 4). This representation allows a visual comparison of the two distributions for the parameter considered.

**Table 1:** Physicochemical parameters showing significant differences between the AP and NAP groups^a^.

**Parameter tested**	**In AP/NAP**	**p-value**
Aromatic amino acids (F, W, Y, H)	less	2.8E-07
Polar amino acids (R, K, E, D, Q, N)	more	1.0E-03
β-strand amino acids (V, I, Y, C, W, F, T)	less	1.4E-03
Hydrophobic amino acids (C, L, V, I, M, F, W)	less	1.6E-03
α-helix amino acids (E, A, L, M, Q, K, R, H)	more	2.2E-03
Basic amino acids (R, K)	more	2.7E-03
Arginine (R)	more	2.7E-03
Neutral amino acids (all except K, R, E, D)	less	3.1E-03
Tyrosine (Y)	less	3.7E-03
Histidine (H)	less	2.1E-02
Phenylalanine (F)	less	2.1E-02
Acidic amino acids (E, D)	more	2.6E-02
Glutamate (E)	more	2.6E-02
Tryptophan (W)	less	2.7E-02

*a* In this Table are reported the most significant parameters (with p-value < 0.05; see Table S2) ordered by increasing p-values, i.e beginning with the most significant ones.

A striking result is the low abundance of aromatic residues (Phe + Trp + Tyr + His) in the APs compared with the NAPs (p-value = 2.8E-7). Remarkably, this bias involves each of the four aromatic amino acids (Table 1), including His, which is also considered as aromatic according to the Hückel rule (see 23). The second important result concerns the charged amino acids Arg and Glu, which are found in excess in the AP subset (Table 1 and Figure 4). This result is consistent with a major importance of electrostatic interactions in the adsorption process. Though no significant difference is observed for the two other charged amino acids, Lys (Figure 4, p-value = 0.41) and Asp (p-value = 0.44), the APs contain an excess of polar residues charged positively as well as negatively (Table 1). Conversely, the APs contain a reduced number of hydrophobic and neutral amino acids. Finally, residues involved in α-helix are enriched in APs and residues involved in ß-strands are enriched in NAPs.

Most of the remaining features analyzed were either not significant (p-value > 0.05). For instance, protein size (p-value = 0.08) and isoelectric point (p-value = 0.53) were not relevant in this study. Though this latter result was somewhat surprising, we noted that it is consistent with previous data [24]. Hence, though the total number of positively and negatively charged residues seems important for protein adsorption, the net charge of the proteins does not seem relevant in the process.

### Structural analyses

Whereas the importance of charged amino acids for binding could be interpreted in terms of electrostatic interactions (see Introduction), the relevance of aromatic residues in limiting protein binding to NPs was more surprising. Aromatic amino acids have different functions in proteins including folding, protein-protein recognition, ligand binding, catalytic activity and structural stabilization. In order to determine the consequences of the amino acid bias in the AP versus NAP subsets in terms of structures and interactions, we searched, among the two sets, the proteins with known structure. We found respectively 12 and 18 structures from the AP and NAP subsets (the structures are listed in Table S1), which represented 39% of the proteins in each group (12/31 for AP subset and 18/46 for NAP subset). Based on the monomer structures of each protein, we calculated the accessibility of charged residues for both groups. No significant difference was observed between the two groups (data not shown). The ability to bind NPs could not be explained by a difference in positive charge (or negative charge) accessibility at the surface of the proteins. In a second step, we quantified the number of different types of intramolecular interactions in the two subsets (see Table 2). These noncovalent attractive interactions are crucial for maintaining the 3D structure of proteins. Our results clearly show that, except for ionic type (salt bridges), there is a significant increased of the interactions in the NAP group. Proteins of the NAP subset thus appear to be more structured with an increased internal cohesion. Moreover, two of the strongest interactions, namely π-π and cation-π interactions, represent by far the highest augmentation, respectively 77% and 30%, and are well correlated with a higher content of aromatic residues in the NAP subset. This analysis thus shows a clear correlation between the content in aromatic residues, the amount of π-mediated interactions and protein structural stability. We also noted that the 30% augmentation of cation-π interactions occurs despite a shortage of basic amino acids in the NAP group. A consequence of this increased implication of basic residues in cation-π interactions in the NAPs is that the number of positively charged residues remaining free to interact with negatively charged NPs is thus reduced in the NAPs. In the same line, the unchanged amount of ionic interactions in the NAPs in spite of a lower content of basic residues decreases the available positive charges in the NAP proteins. We conclude that the positively charged residues available for interactions with silica tend to be markedly higher in the APs than in the NAPs. 

**Table 2:** Interaction type per amino acid measured in 3D structures in both groups of proteins^***a***^.

**Interaction type**	**Ionic/AA^*b*^ (6Å)**	**Cation-π /AA (6Å)**	**π-π /AA (4.5-7Å)**	**H bond /AA SC^*c*^-SC**	**Hydrophobic /AA (5Å)**	**Total attractive interactions /AA**
AP	0.100	0.02	0.026	0.278	0.785	1.204
NAP	0.102	0.026	0.046	0.325	0.858	1.359
% of increase	2%	30%	77%	17%	9.5%	13%

*a* The mean values were calculated for 12 and 18 protein structures of the AP and NAP groups respectively (see list in Table S1).

*b* AA: amino acid.

*c* SC: side chain.

**Figure 5:**
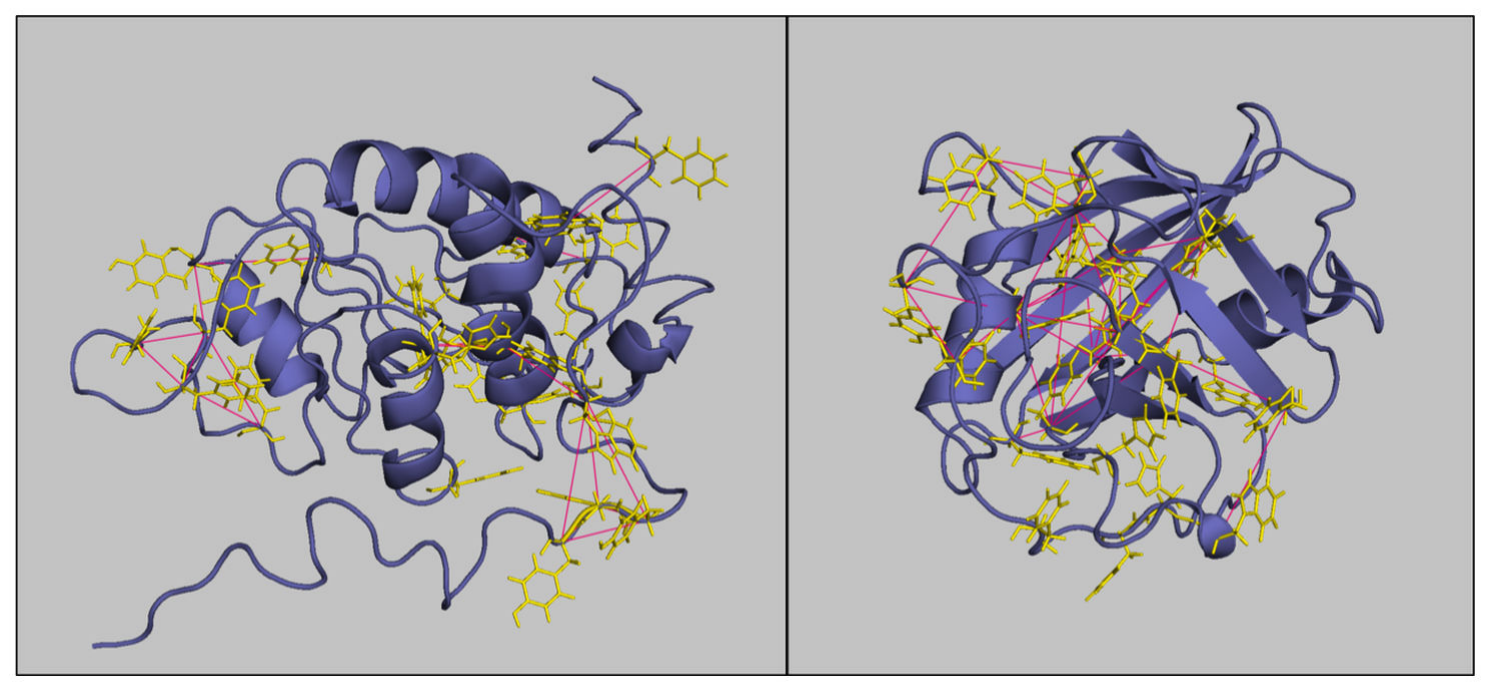
Representative structures of proteins belonging to AP and NAP groups. Left: Rps0a for the AP group. Right: Cpr1 of the NAP group. The secondary structures of the protein are displayed in cartoon mode. The aromatic residues are shown in yellow in line mode and the interactions between backbone carbon atoms of the aromatic residues are displayed in red to show the aromatic clusters in both groups.

The two protein structures presented in Figure 5 are typical of each group. The protein Cpr1 representative of the NAP subset is more structured and enriched in aromatic clusters. The protein Rps0A belonging to the AP subset is more disordered and the aromatic amino acids are less numerous and clustered. 

**Figure 6:**
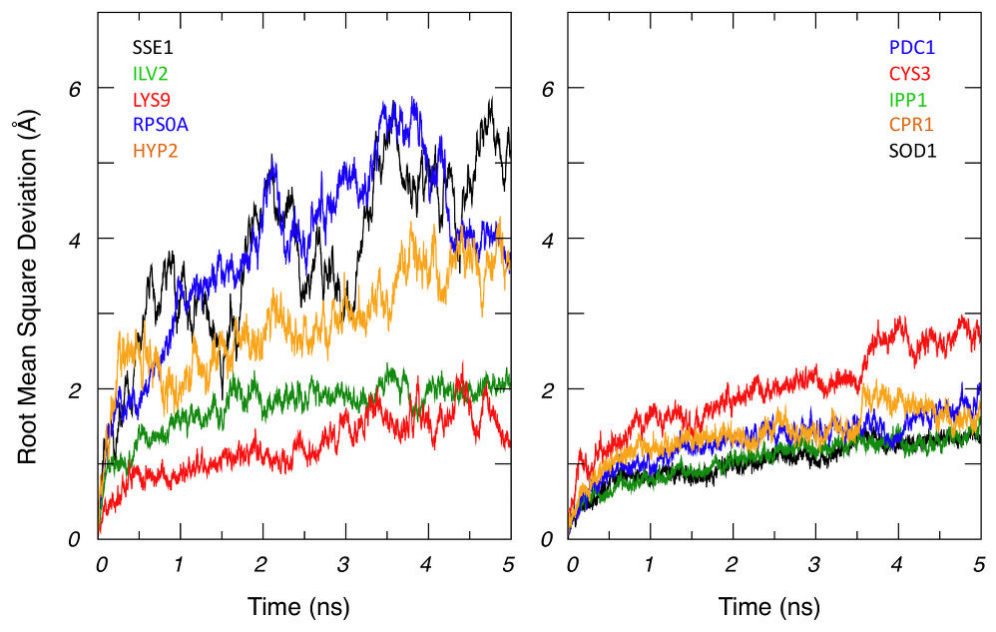
Root Mean Square Deviation calculated from molecular dynamics trajectories. AP group (left) and NAP group (right).

Among these proteins with known structures, we selected in each group the five proteins with the best structural resolution, to analyze their structural stability by molecular dynamics simulations. The idea was to test on a small protein subset whether protein adsorption and protein flexibility were indeed correlated. Figure 6 shows the change in root mean square deviation (RMSD) over the 5 ns production period of the simulation time for the proteins of the two subsets. Three proteins (Sse1, Rps0A and Hyp2) of the AP subset present strong deviations, rapidly reaching an RMSD value of ca. 4 Å while the deviations for the NAPs remain significantly lower (average RMSD value of 1.35 Å). We carefully checked that these large RMSD values were not the result of artifacts such as the energy terms and the temperature. The largest RMSD values could be explained due to specific structural features: the structure of Sse1 is extended and the C terminal part of Rps0A is unstructured, which confer larger flexibility properties for these molecules. For the 3 other proteins (Hyp2, Ilv2 and Lys9) the mean RMSD value remains always significantly higher (1.95 Å) than those of the NAP subset. This is confirmed with the analysis of B-factor per residue, which reflects the local flexibility of the structure. The mean value per residue is 95 for the AP subset whereas it is 33 for the NAP subset (data not shown). From these simulations, we conclude that proteins binding to silica NPs are more flexible than the NAPs. 

## Discussion

### The methodological approach

Omics methods have gained great popularity in cellular biology and biochemistry. However, despite their high accessibility nowadays, they have seldom been used to resolve physicochemical issues. We demonstrate here that proteomics, associated with molecular modeling and rigorous statistical analysis, can help to shed light on a longstanding issue: the physicochemical determinants of protein adsorption/non-adsorption. The method developed and validated in this work consists in sorting, among a pool of proteins, those proteins prone to adsorb silica NPs from those that resist adsorption. If the proteins of the two subsets are sufficiently numerous and varied, the statistical comparison of these two subsets allows the identification of the physicochemical determinants responsible for adsorption and non-adsorption. This strategy has the advantage of throughput compared with most adsorption studies conducted on isolated, purified proteins. It allows to directly compare the adsorption properties of many different proteins under the same physicochemical conditions.

We used an extract of total soluble proteins produced in yeast. The proteome that can be analyzed by 2-DE is composed of more than 100 soluble proteins with isoelectric points ranging from 3.5 to 7.5 and molecular weights from 10 to 120 kDa. Most of these proteins are globular, highly soluble and have metabolic functions in yeast [25]. Their abundance ranges from a few percent of the total soluble proteins (e.g. Tdh3, Eno2) to less than 0.01% (e.g. Bgl2, Kar2) [26]. Among the yeast proteins, a high number (31 for the APs and 46 for the NAPs) clearly behaved differently with respect to silica. The size of these groups permitted a statistical comparison and the determination of the physicochemical parameters essential for adsorption and non-adsorption. Though many studies have analyzed and listed the proteins adsorbed on NPs in blood plasma (for review see 8), the approach developed here has, to our knowledge, never been used. We noticed, however, the study of Tenzer et al. [24], which showed that, in accordance with our data, protein charge and protein size do not correlate with protein adsorption/non-adsorption. Unfortunately, this study did not analyze other protein parameters such as amino acid composition. The problem of plasma protein pools is their high dynamic range in terms of protein abundance, i.e. the 10 proteins the most abundant represent more than 99% of the total bulk mass of proteins present in the plasma [27]. In the best case, even with an efficient LC/MS/MS analysis, the number of proteins analyzed are too low to constitute groups of sufficient size and thus to determine relevant criteria for protein adsorption. This difficulty emphasizes the advantage of using an extract of yeast proteins, which presents a lower dynamic range of protein abundance. The analysis can also be deepened by the use of a labeled pool of proteins. Using a pool of ^35^S-labeled proteins facilitates the visual and quantitative analysis of the data even with low amounts of protein on the gels. In such a way, we could routinely analyze about 100 proteins. Note that the method proposed in this paper can be applied to any other type of surface. A possible drawback of the method is that we cannot exclude that some of the proteins identified as adsorbed on the NPs are in fact proteins with a strong affinity for a true AP protein. However, this occurrence should be rare since our search in the yeast databases retrieved only 3 physical interactions between members of the AP subset.

### Importance of charged amino acids

An important result of this analysis is that the net charge of the proteins is not a distinctive criterion among the two groups. However, the total number of negatively and positively charged amino acids is markedly increased in the AP subset (p-value = 2.6E-2 and 2.7E-3, respectively), meaning enhanced charge density, polarity and hydrophilicity. A direct consequence is the presence of an increased number of positive charge clusters in the AP subset (Figure S1), which may help to initiate locally a series of electrostatic interactions with the SiO- groups. This interpretation is consistent with molecular dynamics simulations of the initial steps of lysozyme interaction with the silica surface [28], suggesting a major importance of several positive charges (mainly Arg residues) in a small area of the protein. Interestingly, the analysis of the 3D structures (Table 2) showed that the number of basic residues (Lys and Arg) not involved in intra-molecular interactions (salt bridges or cation-π) and thus available for electrostatic interactions with the silica surface is higher in the APs than in the NAPs. This effect concerns mainly Arg, which is preferred to Lys in salt bridges [29,30] and in cation-π interactions [29,31] probably contributes to increase the differences between APs and NAPs in the probability of electrostatic interaction with the silica surface.

Among basic amino acids, only Arg residues are over-represented in APs, with no bias in favor of Lys and His. This significant difference between Arg and Lys is quite unexpected as one generally assumes that these two basic amino acids have a similar capacity for electrostatic interactions. The difference may arise from the specific interaction of Arg with silica. When we consider the free amino acids, Arg adsorbs better to nanosized silica than His, Lys, and ornithine [32]. One can suppose that the guanidinium group (specific to the Arg residue), with its delocalized positive charge on three nitrogen atoms, may have a higher probability of establishing an electrostatic interaction with the silica surface. Consistently, molecular dynamics simulations of lysozyme adsorption on a silica surface show that Arg residues play a greater role than Lys residues [28]. One hypothesis, already proposed by Hoefling et al. [33], is that Arg, more than Lys, facilitates the initial contact(s) as it is more prone to penetrate the water layer, and is able after a first contact to change the conformation of the charged guanidinium in order to enhance the interaction surface. In addition, the charged guanidinium group can potentially establish more hydrogren bonds than lysine side chain with the OH groups of the silica surface [34,35]. Arg would thus, more than Lys, be able to deal with the spatial constraints to establish stronger interactions with silica.

The enrichment in Glu residues in the AP subset may be considered as counter-intuitive: unlike Arg, Glu is negatively charged at pH 7.4 and the binding of this amino acid to the electronegative silica surface is therefore unexpected [36]. We thus suppose that Glu does not bind directly to the silica surface, though this hypothesis cannot be totally excluded [37]. An interpretation is that positive cations such as Na^+^ cations present at high concentration (150 mM) in the PBS buffer used in our study would be attracted by the silica surface [35] and would screen at least partially the electronegative surface, favoring the attraction of glutamate residues in some places [1]. Another explanation is to consider that the Glu bias is linked to the overall enrichment in both negatively and positively charged residues in the AP subset. In other words, the excess in Glu residues would participate in the electrostatic compensation for the excess of Arg in the AP subset.

### “Hard” proteins are more resistant to adsorption

Our work shows a close correlation between 4 properties of proteins: (i) their aromatic residue content, (ii) their amount of π-π interactions, (iii) their level of structural stability (hardness) and (iv) their capacity to resist adsorption. These correlations have been suggested by both statistical comparison of the primary sequences and structural analysis of APs and NAPs. They were further confirmed by analyzing the known 3D-structures of proteins from the 2 groups and by molecular dynamics simulations. 

The notion that protein plasticity is an important determinant in the adsorption process is not new, but strong evidence demonstrating this concept is still lacking, though a body of arguments and data supports this notion [15-18]. An interesting example is the α-lactalbumin, for which the less stable form (Ca^2+^-depleted) has a higher affinity for silica surfaces than the stable form (Ca^2+^-replete) [38]. In this context, our data showing that APs are significantly less structured and more flexible than NAPs help strengthen the above hypothesis.

### Importance of π-π interactions in protein “hardness”

The origin of protein structural stability is quite controversial. Usually, hydrophobic cores (along with hydrogen bonds and salt bridges) are thought to be a major determinant of structural stability [39-42]. The amino acids classically considered to be mainly involved in the hydrophobic cores are Leu, Ile and Val. Aromatic residues such as Phe and Trp are also considered, but are of lesser importance because of their lower abundance in proteins. In addition, aromatic residues (in the IUPAC definition of the word) like His and Tyr are usually not considered in the hydrophobic core. Alternatively, other studies suggest a major importance of aromatic residues in the hydrophobic core during the first steps of protein folding [43-45]. It has also been shown that protein structural stability is strengthened by “pure” π-π interactions [46,47] and by multiple interactions constituting spatial clusters of aromatic amino acids [48-50]. In our study, the impact of classical hydrophobic amino acids seems limited. In the proteins of known structure, the occurrence of hydrophobic interactions was increased by 9.5% in NAPs compared with APs (Table 2), whereas the increase in π-π interactions (involving Phe/Trp/Tyr/His) reached 77%. Moreover, another argument supporting the preeminence of aromatic interactions over hydrophobic interactions is that, among the aromatic residues, the strongest bias was not for the hydrophobic Phe and Trp but for His and Tyr, which do not have hydrophobic properties. Thus, beyond the question of “to bind or not to bind” to silica NPs, our work strongly suggests that aromatic clusters are of major importance in the cohesive core of the proteins and the control of their “softness/hardness”.

## Conclusions

Our study highlights two major determinants of adsorption/ non-adsorption on silica surface: (i) electrostatics, with an important role of arginine residues and (ii) protein flexibility, partly controlled by aromatic residues. These two characteristics are key determinants in the two steps of the rough adsorption model depicted in Figure 1. Our data strengthen the notion that aromatic clusters (π-π interactions) are of major importance for maintaining the rigid structure of “hard” proteins, which prevents their spreading on the surface. The “soft” proteins, less rich in aromatic residues, are more prone to deform and to establish an increased number of electrostatic interactions and hydrogen bonds between the protein and the surface. Alternatively, during the spreading process, some apolar amino acids normally buried inside the protein core become exposed outside, favoring hydrophobic interactions with siloxane sites (-Si-O-Si-) [17]. In terms of thermodynamics, the driving force would be mainly enthalpy in the first step and entropy in the second step, with the removal of surface-bound water molecules and salt ions and with the structural rearrangements of the protein. 

In this work, we proposed a strategy that we validated with silica NPs. The method can be used to study protein interactions with other different types of surfaces (e.g. with other chemical compositions or other sizes). We must keep in mind that the data shown in this work were obtained with NPs for which we expect quasi uniform surfaces properties.  For micro-, meso- and macro-materials, the surface charge pattern can be less homogeneous, which may lead to a variable importance of  the role of the charged amino-acids from one surface to another. On the other hand, the role of protein softness in enabling deformation and maximizing interactions (of any type) with surfaces is expected to be a general phenomenum, irrespective of the surface electrostatics or of its geometrical characteristics.

Overall, this study validates the proof of concept of the proposed strategy, deepens our understanding of protein adsorption on SiO_2_ surfaces and opens the way of using these parameters (arginine and aromatic content in proteins) to develop models predicting adsorption/non adsorption.

## Materials and Methods

### Silica nanoparticle characterizations

Silica nanopowder (SiO_2_) of 99.5% purity was from Sigma-Aldrich (637238) (CAS Number 7631-86-9). According to the manufacturer, the NPs are 10-20 nm in size and have a specific surface area of 140-180 m^2^/g. NPs were prepared in phosphate buffer saline (PBS) pH 7.4 at the final concentration of 5 g/L and characterized by TEM, SANS, zetametry and gas adsorption isotherms. 

TEM images were recorded at IMAGIF (Centre de Recherche de Gif, CNRS) on a Jeol JEM-1400 transmission electron microscope operating at 60 kV and equipped with an Orius SC1000 camera. 3 µL of 0.5 mg/mL NP solution was deposited on an ionized 400 mesh carbon-coated Cu grid and left to evaporate for 5 min. Particle size distribution was obtained by image analysis with ImageJ software. Surface area was measured using nitrogen adsorption-desorption isotherms at 77K recorded on a Micromeritics apparatus (ASAP 2010 Instrument). Samples were prepared by drying 1 g of NPs under vacuum at 90°C for 1 h and at 105°C for 2 h to ensure complete moisture desorption before analysis. The specific surface area, noted S_BET_, was calculated as between 0.06 to 0.2 P_00_ using the Brunauer-Emmet-Teller method [51]. Zeta potentials of NP solutions (0.1 mg/mL) were measured in buffered solutions on a Malvern Zeta Nanosizer. Zeta potentials were calculated by fitting the electrophoretic mobility to the Smoluchowski model, which is valid for aqueous solutions with medium electrolyte concentrations [52].

SANS experiments were performed on a PAXY small-angle spectrometer of the ORPHEE facility at Laboratoire Léon Brillouin. Small-angle neutron scattering spectra were recorded at room temperature using dry NPs in a 1 mm quartz cell according to two different setups defined respectively by a sample-detector length of 5 m and 2 m and a selected wavelength of 9 Å and 6 Å. Overlapping of wavenumber transfer q ensures correct absolute values for the scattered intensities measured and lack of multiple scattering. Data were processed according to [53] to determine the size distribution of the particles.

### Production of the protein mixture

The protein mixture used in this study was produced by the yeast (*Saccharomyces cerevisiae*) strain S288C (*Matα SUC2 mal mel gal2 CUP1*) [54]. Cells were grown with shaking at 30°C in 1 L of a synthetic defined (SD) yeast medium (6.7 g/L yeast nitrogen base (with (NH_4_)_2_SO_4_) and 20 g/L glucose) to obtain a cellular concentration of 2 x 10^7^ cells/mL. Cells were collected by centrifugation, resuspended in phosphate buffered saline (PBS) buffer containing 5% glycerol and a cocktail of protease inhibitors (complete, EDTA-free from Roche and 1 mM PMSF) and broken using a French press. The extract was centrifuged (14,000 rpm, 30 min, 4°C) and the supernatant containing soluble proteins was recovered. The protein concentration was determined by the method of Bradford [55]. Typically, 1 L of yeast cells yielded 4 mL of soluble proteins at about 20 μg/μL.

To produce ^35^S-labeled proteins, 50 mL of the unlabeled yeast cell culture (see above) was centrifuged, resuspended in a medium devoid of sulfate for 30 min and the cells were labeled with 1 to 2 mCi of ^35^S-methionine for 30 min. Under these conditions, nearly all the ^35^S-methionine is used for protein synthesis and the amount of ^35^S accumulated in ^35^S-metabolites is less than 1% (data not shown). The proteins were prepared by the same protocol as for the unlabeled protein mixture except that the volumes were ten-fold lower and that the cells were broken with beads instead of the French press. In typical experiments, we obtained about 0.5 mL of soluble ^35^S-protein extract at a concentration of 2 μg/μL containing about 2 μCi/μL.

### Mixing proteins and SiO_2_ nanoparticles

The stock solution of dispersed SiO_2_ NPs was prepared in PBS buffer pH 7.4 at the concentration of 25 g/L. The NPs were diluted in the protein solution to give a final concentration of 5 g/L, representing a surface of 0.168 m^2^ for adsorption per experiment (in a final volume of 0.2 mL). The protein solution was a mixture of unlabeled and ^35^S-labeled yeast proteins at the relevant protein concentrations. The ^35^S-labeled proteins were used as a tracer in the isotherm experiments and two-dimensional electrophoresis (2-DE). 

For the isotherm experiments, the final protein concentration ranged from 0.15 g/L to 6.5 g/L. The mixtures of proteins and NPs were incubated at room temperature for 3 hours with gentle shaking. NPs (with adsorbed proteins) and non-adsorbed proteins were separated by centrifugation (4000 rpm, 2 min, 20°C). The NPs (with adsorbed proteins) were washed in PBS buffer and counted by liquid scintillation (liquid scintillation counter Wallac 1409 DSA). Preliminary tests showed that no radioactive quenching was induced by the presence of SiO_2_ NPs in the counted samples. 

For the other experiments, the final concentration of unlabeled + labeled proteins was 1 g/L. At this concentration, 37% of the proteins are adsorbed and 63% remain in solution. The adsorbed protein fraction and the non-adsorbed protein fraction were prepared as previously and processed for 2-DE.

### Two-dimensional gel electrophoresis

Proteins of the supernatant were concentrated 5-fold by evaporation and 4 µL of the concentrated solution was added to 16 μL of 2-DE buffer [DNase 1 (100 U/mL), Rnase A (10 U/mL), CaCl_2_ (20 mM), MgCl_2_ (50 mM), TrisHCl pH 7 (100 mM), SDS 0.02%, ampholine (1%), CHAPS (0.8%), β-mercaptoethanol (1%) and urea (8 mM)]. After incubation for 15 min at 30°C, 0.5 µL of bromophenol blue was added and the sample was processed on 2-DE as described in [25].

NP pellets containing the adsorbed proteins, were washed twice with 200 µL PBS buffer and then incubated for 15 min at 30°C in 20 μL of 2-DE buffer. The mixture was centrifuged (13,000 rpm, 3 min) to separate the NPs (in the pellets) and the desorbed proteins (in the supernatant). Scintillation counting of the NPs confirmed that all the proteins bound to the NPs were solubilized in the 2-DE buffer. Bromophenol blue (0.5 µL) was added to the supernatant and the sample was run on 2-DE gels.

The typical quantity of protein used for the 2-DE gels was about 50 μg for adsorbed proteins and about 10 μg for non-adsorbed proteins. This value (10 μg) was limited because the high salt concentration (PBS buffer) in the non-adsorbed protein extract perturbs the isofocusing electrophoresis. After electrophoresis, gels were stained with Coomassie Blue R-250, dried, and processed for autoradiography by standard procedures. As expected, exposure times necessary to obtain comparable image intensities were 5-fold higher for non-adsorbed proteins than for the adsorbed proteins. The ^35^S images of the gels were also recorded on PhosphorImager (Molecular Dynamics STORM). The experiments were repeated 3 times independently with identical results. 

### Protein identification and mass spectrometry

A 2-DE gel reference map of total soluble yeast proteins is available in the laboratory based on previous identifications by different methods [25] including mass spectrometry [56,57]. Adsorbed and non-adsorbed protein fractions on 2-DE gels were identified essentially by comparing the two corresponding gels with the gel of the total protein extract (see for example Figure 3B, 3C and 3D). When an unknown protein spot appeared on the gel(s) or for some confirmations of previously known spots, identifications were performed by mass spectrometry. Spots of interest were extracted from non-radioactive gels and the proteins were digested in-gel by trypsin using standard protocols [58]. Spot extracts were resuspended in 5% acetonitrile, 0.1% formic acid (buffer A). Peptide extracts were submitted to nanoLC-MS/MS using an Ultimate 3000 nanoLC system (Dionex, Thermo Fisher Scientific) connected to an ESI-LTQ-Orbitrap mass spectrometer (Thermo Fisher Scientific) equipped with a nanoelectrospray ion source. The ion spray voltage was set at 1.4 kV with a transfer capillary temperature of 200°C. Five microliters of each sample was loaded on a C18 precolumn (300 µm inner diameter x 5 mm, Dionex), and desalted for 5 min at a flow rate of 20 µL/min with 100% buffer A. Then, the precolumn was switched connected to an analytical capillary C18 column (75 µm inner diameter x 15 cm; AcclaimPepMap 100, Dionex, Thermo Fisher Scientific) equilibrated in 100% solvent A. Peptides were eluted using a 0-70% gradient of solvent B (80% acetonitrile, 0.1% formic acid) over 50 min at a flow rate of 300 nL/min. The mass spectrometer was operated in the data-dependent mode to automatically switch between Orbitrap MS and LTQ-MS/MS acquisition. Survey full scan MS spectra were acquired in the Orbitrap in the 400-1600 *m/z* range with the resolution set to a value of 60,000 at *m/z* 400. Sequential isolation of the 5 most intense precursor ions was carried out, followed by their fragmentation by collision-induced dissociation in the linear ion trap.

 Data were analyzed using the locally installed Mascot search engine (version 2.2.1, Matrix Science). Peak lists were searched against *S. cerevisiae* sequences in the Swiss-Prot database. Mass accuracy was set to 10 ppm for MS mode and 0.6 Da for MS/MS mode and one missed tryptic cleavage site was allowed in the search. Oxidation of methionine was searched for as a variable modification and carbamidomethylation was set as a fixed modification.

### Quantitative protein analysis

Radioactive images of 2-DE gels of total protein extract, adsorbed proteins and non-adsorbed proteins, were analyzed using the software Progenesis Samespots v3.0. The automatic matching of the 3 gels by the software was checked, spot by spot, and corrected if necessary. The intensity (volume value) of each protein spot was normalized to the total intensity of the gel. This normalization allows a direct comparison of the relative intensities of each protein spot in the different gels. Using the quantitative data of volume intensity provided by the software, we calculated for each protein the ratio of relative intensity in the gel of the adsorbed proteins to the relative intensity in the gel of the non-adsorbed proteins. High ratios correspond to adsorbed proteins (APs) and low ratios to non-adsorbed proteins (NAPs). To classify the proteins into 3 groups (adsorbed, non-adsorbed and intermediate), we arbitrarily chose the values 10^(0.2)^ = 1.58 and 10^(-0.2)^ = 0.63 as thresholds. If the ratio was higher than 1.58, the protein was classified as adsorbed; if the ratio was lower than 0.63, the protein was classified as non-adsorbed; and if 0.63 < ratio < 1.58, the protein was put in the intermediate group.

### Protein primary structure analysis

Protein coding sequences were extracted from the S. *cerevisiae* full genome version sacCer2 released by UCSC. Statistical analysis was conducted using the R software [59]. 

Structural and physiochemical features of proteins were calculated using Profeat [60] or in specific cases using our own implementations (length, molecular weight, isoelectric point, aromatic amino acid composition). The statistical differences among features between the group of adsorbed proteins and the group of non-adsorbed proteins were exhibited using the Brunner-Munzel test [61] as implemented in the R lawstat package [62]. The non-parametric Brunner-Munzel test is used to assess the stochastic equality of two samples (HO: P(X < Y) = P(X > Y) *against* H1: P(X < Y) <> P(X > Y)). This test is commonly known as a "generalization of the Wilcoxon-Mann-Whithney test" for heteroscedastic cases (i.e. unequal variance or shape distribution). Indeed, we cannot use a student t-test because 40% of the features distributions, such as hydrophobic or neutral amino acids composition, do not fulfill the Gaussian distribution assumption (assess using the robust Jarque-Bera test of normality [63]. We cannot either use the Wilcoxon-Mann-Whithney test because 11% of the features distributions, such as the arginine residue composition, do not fulfill the homoscedasticity assumption (assess using the robust Brown-Forsythe test of equality of variance [64]. Nevertheless, using the Wilcoxon-Mann-Withney test leads to similar conclusions as shown in the Table S2. Resulted p-values were adjusted for multiple comparisons, by controlling the false discovery rate, using the Benjamini-Hochberg method [65].

We also calculated positive (respectively negative) charge profiles, where positively (respectively negatively) charged amino acids are counted over a sliding window of twelve amino acids. The results were filtered by only keeping counts above a threshold of three positively (respectively negatively) charged amino acids per sliding window. To compare profiles between the AP and NAP groups, for each protein we calculated the ratio of the filtered charged window over the total number of sliding windows (denoted charge clusters hereafter). Then charge profile distributions were compared using the same Brunner-Munzel statistical test as previously described.

### Structural analyses

All simulations were performed using the AMBER 9 program suite [66] with the Parm99SB force field. From the 30 structures of proteins that were analyzed, 28 of them were imported from the Protein Data Bank [67] and 2 from the Protein Model Portal [68]. These two models are considered as reliable thanks to the high sequence identity between the template structure and the protein of interest (62% for Krs1 and 69% for Sam1). All the calculations were performed on monomer forms. Hydrogen atoms were added with the LEaP program to complete the X-ray structures. Proteins were then neutralized with Na^+^ cations and immersed in a water box with at least 10 Å-deep solvation shell using the TIP3P water molecules. The structures were then minimized and used to initiate molecular dynamics. All simulations were performes in the isothermal isobaric ensemble (P = 1 atm, T = 300 °K), regulated with the Berendsen barostat and thermostat [69], using periodic boundary conditions and Ewald sums for treating long range electrostatic interactions [70]. The hydrogen atoms were constrained to the equilibrium bond length using the SHAKE algorithm [71]. A 2 fs time step for the integration of Newton's equations was used. The nonbonded cutoff radius of 10 Å was used. All simulations were run with the PMEMD module of the AMBER package.

Molecular dynamics simulations of 5 ns for the production period, without constraints, were used to provide quantitative information about the stability of the models (root mean square deviation; RMSD) and to determine the flexible regions of the structures (B factor). The structures were analyzed with CMview program version 1.1 [72]. The amino acid accessibilities were calculated with Naccess program version 2.1.1 [73] that use the Lee and Richards method [74].

## Supporting Information

Figure S1 **Cumulative distribution for positive charge clusters (left panel) and negative charge clusters (right panel).** The AP group is represented by a solid line and the NAP group by a dotted line. The electrostatic charge distribution over the protein sequences was evaluated by enumerating the number of positive (respectively negative) charges within a sliding window of 12 amino acids along the polypeptide sequences. A threshold of three charged amino acids per sliding window was used to exhibit positively (respectively negatively) charged clusters for each protein.
(EPS)

Table S1 **List of adsorbed and non-adsorbed proteins.**
(XLSX)

Table S2 **List of features analysed and statistically compared between the AP and the NAP groups.**
(XLSX)
